# All in the Details: A First Assessment for the Viability of Metabarcoding in Diet Composition Analysis of African Wild Dogs (*Lycaon pictus*)

**DOI:** 10.1002/ece3.70526

**Published:** 2024-11-13

**Authors:** Bridget C. O'Connor, Bruce Crossey, Grant Hall, Andre Ganswindt, Carel J. Oosthuizen

**Affiliations:** ^1^ Resolve Evolve, Department of Zoology and Entomology University of Pretoria Pretoria South Africa; ^2^ Mammal Research Institute University of Pretoria Pretoria South Africa; ^3^ UP Stable Isotope Laboratory Mammal Research Institute, University of Pretoria Pretoria South Africa

**Keywords:** faecal DNA, high‐throughput sequencing, prey composition, stable isotope analysis

## Abstract

DNA metabarcoding is a contemporary technique in diet composition studies and stands to fill key knowledge gaps left by traditional diet analysis methods. For endangered species such as the African wild dog (*Lycaon pictus*), the fulfilment of these knowledge gaps presents an opportunity for improved management practices and vulnerability assessments. There are an estimated ~600 African wild dogs remaining in South Africa. These dogs are generally understood to prey upon impala (*Aepyceros melampus*) and other medium‐sized ungulates. Here, we present the first assessment of DNA metabarcoding as a valuable method for diet composition analysis of this highly social carnivore. DNA from faecal samples collected across seven landscape types in the Kruger National Park (KNP) was extracted and used to determine the presence of seven unique prey taxa, including novel species such as the Cape hare (*Lepus capensis*). Impala was identified as a prey item in all landscape types, complementing the diet preference prediction made with stable isotope analysis using the same samples and existing understanding of wild dog diet. Given recommended improvements, the application of DNA metabarcoding in wild dog diet analysis shows promising prospects for identifying novel prey species and validating previous records of this endangered canids diet.

## Graphical abstract

This study provides the first results on the diet of the African Wild dog obtained through DNA metabarcoding of faecal samples. Impala was identified as a key prey item which supports existing literature on the species. Smaller mammal species such as the Cape hare, francolins and vlei rats were also identified which may indicate more opportunistic and solitary feeding behaviours not previously observed for this highly social carnivore.

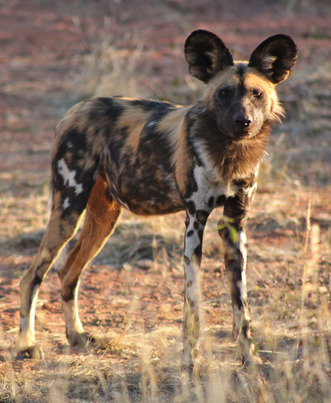

## 1 Introduction

Diet analysis studies play a critical role in understanding the ecology and behaviour of a species (Castle et al. 2020; Jordan 2005; Wang et al. 2022). Knowledge of the diet of certain animals often relies on assumptions based on generalities and ignores differences based on species, ecology, sex and age (Jordan 2005). As well as posing significant knowledge gaps, this has implications for wildlife‐conservation management strategies and the nutritional protocols that may be implemented underneath them (Castle et al. 2020; Jordan 2005). The specific analysis of predator diet composition can function as an indicator of ecosystem health at a larger scale (Wang et al. 2022), highlight limiting factors to the survival of a predator species and identify avenues of competition between co‐existing predators (Morin et al. 2016). Diet analysis studies have also proven useful in conflict mitigation between humans and wildlife. For example, Voigt et al. (2014) demonstrated that cheetah diet on Namibian farmland contained minimal traces of livestock and trophy species.

Traditional methods of diet composition analysis include the visual analysis of faecal matter (Klare, Kamler, and Macdonald 2011), direct analysis of stomach contents (Azevedo et al. 2006) or the real‐time observation of hunts and kill sites (Morin et al. 2016). These methods tend to have limitations in their logistical feasibility and reliability (Carss and Parkinson 2009; Davison et al. 2002; Kohn and Wayne 1997). These limitations have resulted in the application of contemporary stable isotope analysis (SIA) and DNA metabarcoding in determining predator diets, yielding more reliable and accurate predictors than their traditional counterparts (Whitaker et al. 2019).

Dietary studies using stable isotope analyses rely on the differential ratios of stable isotopes (usually ^15^N/^14^N and ^13^C/^12^C) present in taxa at different trophic levels to resolve the diet composition of a chosen study species (Hardy et al. 2010). Carbon isotope ratios are useful in distinguishing between photosynthetic sources in an animal's diet, for example between aquatic phytoplankton or terrestrial plants, C_3_ or C_4_ grasses, or between habitats with sufficiently contrasting vegetation and soil types (Carreon‐Martinez and Heath 2010). Analysis of nitrogen isotope ratios is useful in examining trophic interactions, as the heavier stable isotope (^15^N) is more readily assimilated into the body when food is consumed, while the lighter stable isotope (^14^N) is more readily excreted (Carreon‐Martinez and Heath 2010). Organisms at higher trophic levels are expected to have a larger ^15^N/^14^N ratio upon assessment of their faecal or hair samples (Jeanniard‐du‐Dot et al. 2017). Stable isotopes such as carbon and nitrogen undergo isotopic discrimination when assimilated into tissue such as muscle or keratinised structures like whiskers, hair or nails, where the relative abundance of prey isotope ratios naturally varies between organisms as a result of species‐specific differences in metabolism (Boecklen et al. 2011). Consumers integrate the isotopic ratios of their respective food sources into their tissues, and this in turn can be used to analyse consumer diet composition at the trophic feeding level (Boecklen et al. 2011). Nitrogen stable isotope ratios provide information about the trophic level at which an animal is foraging, and, for predators, respective carbon ratios provide information about preferences between grazing or browsing prey species (Ben‐David, Flynn, and Schell 1997). While SIA is effective in identifying unexpected trophic interactions, dietary overlaps in prey species restrict conclusions on specific trophic interactions, and often resolution of prey species is limited to the feeding guild (i.e., browser, grazer or mixed feeder‐level) (Monterroso et al. 2019).

DNA metabarcoding is the process of identifying the origins of DNA in a specific environmental sample through the use of universal primers that can amplify the DNA of multiple species (Taberlet et al. 2012). The specific amplified sequences resulting from these universal primers are termed ‘barcodes’ which are unique to individual species. These unique sequences are then compared to a reference database of genomes in order to match the unknown environmental DNA to their species of origin (Taberlet et al. 2012). Analysis by DNA metabarcoding provides a more refined and specific view of predator diet composition, often down to species level, and relies less on expert field experience (Kress et al. 2015; Shao et al. 2021). In addition, large numbers of samples can be examined in one sequencing run with high throughput sequencing (HTS), which allows efficient diet composition analyses (Galan et al. 2018). Taberlet et al. (2012) differentiate DNA metabarcoding from standardised barcoding. DNA metabarcoding uses usually degraded environmental DNA (eDNA) to detect many species and thus has greater ecological and conservation applications than standardised barcoding, which relies on long reads of high‐quality DNA and is limited in the number of species which may be identified in a single sample. Taxonomic inferences made using faecal samples and DNA metabarcoding do, however, rely on an existing database of DNA sequences for the prey species of the predator (Bucklin et al. 2016; Taberlet et al. 2012) as well as the accurate design of universal primers used to amplify the ‘barcodes’ of these species (Coissac, Riaz, and Puillandre 2012). For many predator species, existing universal primers are well established and commonly used, some of which have been designed with reference to the whole mitochondrial sequences of almost 800 vertebrate species (Wang et al. 2022).

Recently, studies investigating diet composition across a variety of taxa have utilised both SIA and DNA metabarcoding in order to gain more comprehensive understandings of an animal's diet (Cordone et al. 2022; Hoenig et al. 2022; Soininen et al. 2014). For more cryptic, smaller or elusive species these non‐invasive techniques are useful alternatives to traditional diet analysis methods (Whitaker et al. 2019). The complementary nature of these techniques comes as DNA metabarcoding offers finer insight into stable isotope results. Used in conjunction with one another, SIA and DNA metabarcoding can offer better identification of specialist foraging groups and when used correctly, give insight into broader spatial and temporal differences in dietary preferences (Jeanniard‐du‐Dot et al. 2017). There are, however, caveats to be considered when combining the two techniques. Metabarcoding dietary analysis can only be done with faecal or gut content samples, while SIA can be performed with an additional number of biological sample types (e.g., hair, blood, muscle, bone). Comparisons between sample types can be useful, but hair samples and faecal samples differ in the spatio‐temporal dietary information that they can communicate, specifically longer‐term information from hair samples, but shorter‐term ‘snapshot’ information from faecal samples.

The African wild dog (*Lycaon pictus*), hereafter referred to as wild dog, has been listed as endangered on the IUCN red list since 2012 (Woodroffe and Sillero‐Zubiri 2020). Wild dogs historically occupied most of sub‐Saharan Africa, but their range has since contracted by an estimated 93% to a few local populations mostly falling within southern and eastern Africa (Wolf and Ripple 2017). The ~6600 individuals still distributed across Africa are concentrated in 14 countries, where only half of these countries support potentially viable populations of eight or more wild dog packs (Nicholson et al. 2020). South Africa is estimated to have ~600 wild dogs (Wild Dog Advisory Group 2022) which are spread across three defined population groups. These comprise (1) the managed metapopulation, (2) the free‐roaming population and (3) the Kruger National Park (KNP) population (Tensen et al. 2019).

The KNP wild dog population is the largest in South Africa, with a reported 418 dogs (Wild Dog Advisory Group 2022) and is generally considered unmanaged and a viable population without human intervention (Tensen et al. 2019). Previous investigations into wild dog dietary preferences, conducted through visual analysis or observed hunts, have found impala to be their key prey species (Creel and Creel 1995; Davies‐Mostert, Mills, and Macdonald 2013; Mills and Gorman 1997). Crossey et al. (2021) obtained and measured the stable isotope ratios for hair, whisker and faecal samples from 38 wild dog packs across different landscapes in the KNP and presented the stable isotope dietary analysis results for the hair and whisker samples only. The authors found that small browsers contributed to a larger part of the wild dog diet than previously thought, particularly in Malelane mountain bushveld and thicket (Crossey et al. 2021). They concluded that due to differing hunting strategies and prey abundances, wild dog diet shifted towards smaller browsers in dense habitats (Crossey et al. 2021).

The present study aims to utilise the faecal samples not used by Crossey et al. (2021) to prove the validity, and provide the first assessment of, metabarcoding diet composition analysis in wild dogs. The previously measured stable isotope ratios for the faecal samples are also modelled as a reference for the metabarcoding analysis and to better provide commentary on the viability of DNA metabarcoding in resolving the prey preferences of wild dogs.

## 2 Materials and Methods

### 2.1 Study Site

**FIGURE 1:**
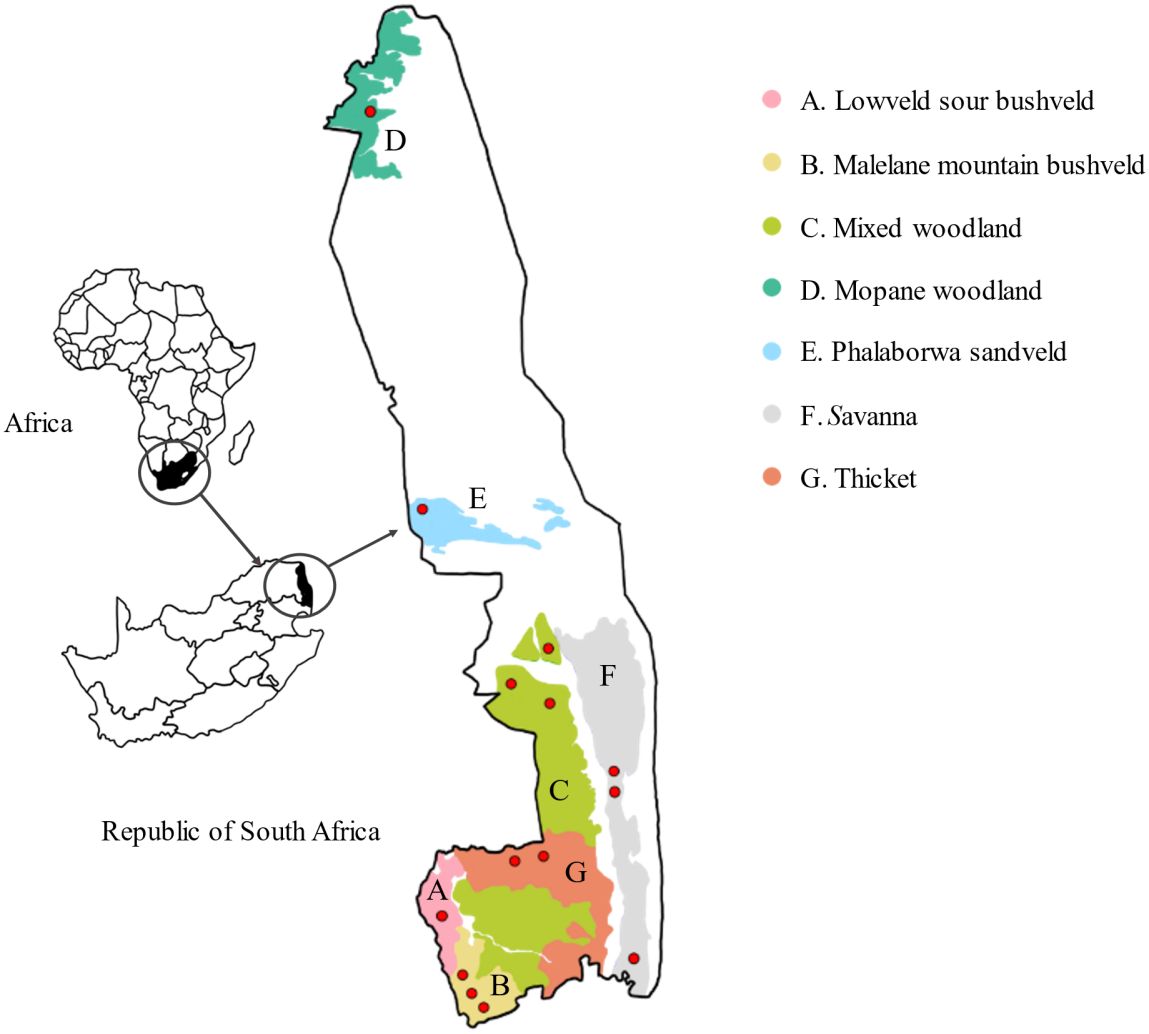
Map showing rough sampling locations (red dots) and respective landscape types, each represented by a different colour according to the insert, within the KNP where wild dogs were sampled. Maps of South Africa and Africa are provided for visual context of where the KNP is situated.

The KNP spans ~20,000 km^2^ of South Africa, situated in the north‐eastern part of the country (Figure 1). The park falls within the provinces of Limpopo and Mpumalanga and is considered a part of the savanna biome (Gertenbach 1983). Most of the KNP wild dog packs inhabit the southern lowveld bushveld zone of the Park (Bothma 2004). Landscape types across the KNP are divided into 35 categories (Gertenbach 1983), but the collected samples originate from only seven of these landscapes (Figure 1); Malelane mountain bushveld, Lowveld sour bushveld, thickets of the Sabie and Crocodile River (hereafter referred to as thicket), mixed *Combretum*/*Terminalia sericia* woodland (hereafter referred to as mixed woodland), *Sclerocarya birrea caffra*/*Acacia nigrescens* savanna (hereafter referred to as savanna), Mopane woodland and Phalaborwa sandveld (the northernmost landscape type).

### 2.2 Stable Isotope Analysis

All research was conducted with the approval of the University of Pretoria Animal Ethics Committee (AEC reference number: NAS048/2023). Faecal samples were collected opportunistically between April 2018 and January 2019 in the KNP under the guidelines of the American Society of Mammalogists (ASM) as per Crossey et al. (2021). Fourteen faecal samples were initially stored at −20°C at the SANParks Veterinary Wildlife Services Biobank and released to Crossey et al. (2021) at the time of their study after being cleared as negative for tuberculosis. Samples were lyophilised from frozen, ground and sieved through a 20 μm metal mesh strainer in sterile laboratory conditions. This was done to remove undigested material from the faecal matter (Fiess, Heistermann, and Hodges 1999). Stable isotope ratios were measured as explained in Crossey et al. (2021) and summarised here; wild dog faecal powder was weighed as aliquots of ~0.4–0.6 mg using a micro‐balance (Mettler Toledo MK5, Mettler Toledo, Columbus, Ohio) and placed into tin capsules which had been precleaned in toluene. Samples were then combusted at 1020°C in an elemental analyser (Flash EA1112 Series), coupled to a Delta V Plus stable light isotope ratio mass spectrometer via a ConFlo IV system (Thermo Fisher Scientific). Two laboratory running standards and a blank sample were run after every 11 unknown samples (Merck Gel and DL‐Valine). These respective running standards are calibrated against international standards (IAEA‐CH‐3, IAEA‐CH‐6, IAEA‐CH‐7, IAEA N‐1, IAEA N‐2, IAEA NO‐3) produced by the International Atomic Energy Association (IAEA) and NBS22 (produced by the US National Bureau of Standards). The precision for δ^13^C was < 0.06‰ and < 0.05‰ for δ^15^N. All results are referenced to air for nitrogen isotope values and Vienna Pee Dee Belemnite for carbon isotope values (Bond and Hobson 2012). Results are expressed in delta notation using a per mille scale (‰) by applying the following standard equation (Coplen 2011):

$$\mathrm{\delta X}=\left[\left(R_{\text{sample}}/R_{\text{standard}}\right)-1\right]$$

where X = ^15^N or ^13^C and R represents ^15^N/^14^N or ^13^C/^12^C, respectively.

### 2.3 Statistical Analysis of Stable Isotope Results in R

As part of this study, statistical analysis was conducted using the MixSIAR package (Stock and Semmens 2016) in the software program R (R Core Team 2021). The MixSIAR package uses Bayesian mixing models to analyse biotracer data such as stable isotopes, fatty acids or element concentrations and estimate the contribution of source (prey) data to a mixture (consumer). This package requires three input sources, namely the stable isotope ratio of the consumer (in this case the stable light isotope data from the wild dog faecal samples), source stable isotope values for potential prey groups, and finally diet‐faeces discrimination factors which account for differences in carbon and nitrogen isotope ratios between diet and faeces. Prey isotope data (Table 1) and discrimination factor (Table 2) values were both extracted from Codron et al. (2006) as also used by Crossey et al. (2021). These prey data constitute isotopically distinct (δ^13^C and δ^15^N) values for prey groups made up of KNP prey species, namely large grazers (Burchell's zebra (*Equus burchelli*), buffalo (*Syncerus caffer*), blue wildebeest (*Connochaetes taurinius*), waterbuck (*Kobus ellipsiprymnus*), reedbuck (*Redunca arundinium*) and sable antelope (*Hippotragus niger*)), large browsers (Giraffe (*Giraffa camelopardalis*) and kudu (*Tragelaphus strepsiceros*)), small browsers (Bushbuck (*Tragelaphus scriptus*), steenbok (*Raphicerus campestris*) and grey duiker (*Sylvicapra grimmia*)) and impala, representing mixed feeders. The wild dog source samples were each tagged with the landscape type from which they were collected (Figure 1), and this was incorporated as a variable in the mixing model.

**TABLE 1:** Carbon and nitrogen stable isotope data for three prey groups and impala (*Aepyceros melampus*) commonly found in the Kruger National Park, based on trophic ecology and body size. From Codron et al. (2006).

Prey species	Faecal samples (*n*)	Converted muscle estimates			
		δ^13^C_VPDB_ (‰)		δ^15^N_AIR_ (‰)	
		Mean	SD	Mean	SD
Large browsers^a^	108	−24.3	0.8	6.6	1.4
Small browsers^b^	47	−23.6	2.3	6.7	1.3
Large grazers^c^	321	−11.3	1.1	5.9	2.1
*Aepyceros melampus*	366	−17.1	3.1	8.0	2.1

^a^ Giraffe (*Giraffa camelopardalis*), kudu (*Tragelaphus strepsiceros*).

^b^ Bushbuck (*Tragelaphus scriptus*), steenbok (*Raphicerus campestris*), grey duiker (*Sylvicapra grimmia*).

^c^ Burchell's zebra (*Equus burchelli*), buffalo (*Syncerus caffer*), blue wildebeest (*Connochaetes taurinius*), waterbuck (*Kobus ellipsiprymnus*), reedbuck (*Redunca arundinium*), sable antelope (*Hippotragus niger*).

**TABLE 2:** Diet discrimination factors for the stable carbon and nitrogen isotope differences between diet and faeces. From Codron et al. (2006).

Tissue(s)	Carnivores			
	δ^13^C_VPDB_ (‰)		δ^15^N_AIR_ (‰)	
	Mean	SD	Mean	SD
Δ Diet‐faeces	−0.9	0.4	+1.0	0

### 2.4 DNA Extraction and Sequencing

DNA extractions were conducted with 40 mg of faecal sample added to 140 μL of water in a 2 mL Eppendorf (Merck) tube. Extractions were conducted for 14 samples using the DNeasy Blood & Tissue Extraction Kit (QIAGEN) with modifications. Modifications included a one‐minute vortex step before a one‐minute centrifugation step at 14,000 rpm following the three‐hour incubation step at 56°C after the addition of proteinase K (Inqaba Biotech). Following this, the supernatant was used according to the standard kit protocol. The remaining solid faecal sample was stored and used for re‐extraction as required. Re‐extraction was used for nine of the 14 samples, and the same protocol as described above was followed, with repeat additions of reagents and an additional three‐hour incubation step.

Universal vertebrate primers (Anatech) that amplified the 12S and 16S gene regions of the mitochondrial genome, designed by Wang et al. (2022) (Table 3), were used. The primers were designed to amplify a wider set of vertebrate species than previous commonly used primers. These primers are degenerate, meaning some positions in the sequence have several possible bases labelled according to the standard IUPAC nucleotide code. For the polymerase chain reaction (PCR, Saiki et al. 1988), both forward primers for the 12S gene fragment (VertU V12S‐U F1 and Vert U V12S‐U F2) were added in equal volumes. Each amplification reaction was conducted in a total reaction volume of 25 μL. Each 25 μL reaction included: 3.5 mM MgCl_2_, 1 x reaction buffer, 0.25 mM of each of the four deoxyribonucleotide bases (Inqaba Biotech), 0.16 μM each of the 12S or 16S‐forward and reverse primers, 0.75 U of SuperTherm Taq DNA Polymerase (ThermoFisher Scientific), 8 U of BSA (bovine serum albumin, Inqaba Biotech) and 4 μL of DNA.

**TABLE 3:** PCR primer sets used to amplify the 12S and 16S fragments in this study. From Wang et al. (2022).

Gene region	Primer	Fragment length (bp)	Sequence 5′–3′
12S	VertU V12S‐U F1	207	TYG TGC CAG CNR CCG CGG TYA
	VertU V12S‐U F2		GTG CCA GCN RCC GCG GTY ANA C
	VertU V12S‐U R		ATA GTR GGG TAT CTA ATC CYA GT
16S	VertU V16S‐U F	241	ACG AGA AGA CCC YRY GRA RCT T
	VertU V16S‐U R		TCT HRR ANA GGA TTG CGC TGT TA

PCR thermocycling was conducted in a 2720 Thermal Cycler (Applied Biosystems). Polymerase chain reaction cycles consisted of an initial denaturation phase that separated the strands of existing DNA and lasted for 2 min at 94°C. This was followed by 35 cycles of denaturation, annealing and elongation. The denaturation phase lasted for 30s at 94°C, the annealing phase where primers bind to the existing template DNA strands continued for a 20s period at 47°C, then the elongation phase proceeded for 20s at 72°C to allow the primers to extend and synthesise the new DNA strands. Finally, an extended elongation phase lasted for 5 min at 72°C to ensure complete synthesis of all fragments.

PCR amplification success was assessed through electrophoresis using 1.5% agarose (separations) gels using 4 μL of the PCR product. Negative controls, using distilled water in place of DNA, showed no contamination. In order to confirm that the PCR reagents were functional, DNA extracted from the blood sample of a female lion *Panthera leo* was used as the positive control. Replicate PCR reactions were performed, the number of which depended on the PCR amplification success of each reaction for each sample (between 3 and 5 repeats per sample were performed and later combined to increase the concentration of the amplified product to an acceptable level for downstream applications).

After amplification, the PCR products were purified according to the protocol outlined in the IonXpress Plus gDNA Fragment Library Preparation user guide (ThermoFisher Scientific). PCR products were purified using 1.8× sample volume of Agencourt AMPure XP Reagent (Beckman Coulter). Following purification, the concentration of each DNA sample was measured using Qubit Fluorometric Quantification according to the protocol outlined in the Qubit dsDNA HS Assay Kit user guide (ThermoFisher Scientific) and using three microliters of DNA in each sample reading. Replicates were combined such that each sample had a minimum concentration of at least 100 ng/μL of DNA. The 11 samples were not pooled into a single library but were kept separate. Each sample comprised a combination of the 12S and 16S purified products combined in equal concentrations. Library construction and sequencing were conducted by the Central Analytical Facilities, Stellenbosch University, and the Ion Torrent Next Generation Sequencing platform was used for sequencing.

### 2.5 Metabarcoding Bioinformatics

The raw sequenced reads first underwent pre‐processing to filter and correct any errors introduced during amplification and sequencing. Pre‐processing was conducted using the shell script bbduk.sh in Ubuntu, part of the BBTools package (Bushnell 2014). The reads were also trimmed to remove low quality positions based on their phred score (Q20). Primer sequences were removed, and sequences were filtered to a minimum of 80 bp. Processing and post‐processing was then conducted in R (R Core Team 2021) using the open‐source software package DADA2 (Callahan et al. 2016). Sequences were further filtered in R to be a minimum of 150 bp long. During processing, Amplicon Sequence Variants (ASVs) were clustered by grouping sequences based on minimal nucleotide differences, frequently one single nucleotide difference. DADA2 generated a parametric error model trained on each sequencing run to minimise and correct false positive errors, and effectively collapse the sequences into ASVs. Multiple ASVs may identify the same taxon as different markers produce different sequences for the same species that are collapsed into an ASV. Following clustering, taxonomic identification of ASVs was conducted using a similarity‐based method, Basic Local Alignment Search Tool (BLAST), that aligns query sequences with a chosen reference database. A custom reference database was generated, comprising 47 likely prey species from the KNP compiled using sequences from annotated whole mitochondrial genomes present on GenBank (Table A1). The custom database was generated based on existing knowledge of the diet of African wild dog prey species known to inhabit the KNP and preliminary BLAST searches conducted on GenBank. Taxonomic assignment was accomplished using the R package taxonomizr (Sherrill‐Mix 2018) which efficiently assigned taxonomy and accession numbers to taxonomic IDs by providing functions that inspect NCBI taxonomy files and accession dumps. The R package Phyloseq (McMurdie and Holmes 2013) was used to perform further analyses, remove one ASV of overrepresented host species and ASVs with less than 10 reads and construct a taxonomy table. DNA was successfully extracted from 11 of the 14 samples (Table 4) and samples that failed to PCR amplify for both gene regions, after multiple DNA extraction reactions, were excluded from the analysis. Successful amplification was obtained for both the 12S and 16S gene regions in all except one of the samples, WD4, in which only the 16S gene fragment was successfully amplified (Table 4). Table 5 outlines the DNA content (in ng) and the initial number of reads sequenced for the 11 successfully amplified samples, and how many reads were discarded at each stage of the bioinformatic pipeline.

**TABLE 4:** List of samples included (labelled with ‘yes’) in stable isotope analysis (SIA) and metabarcoding analysis (by gene regions successfully amplified). Samples labelled with ‘no’ were not included in the SIA analysis or failed to amplify 12S and/or 16S.

Sample	Landscape	Included in SIA	12S gene region amplified	16S gene region amplified
WD1	Malelane mountain bushveld	No	Yes	Yes
WD2	Mixed woodland	Yes	Yes	Yes
WD3	Mixed woodland	Yes	Yes	Yes
WD4	Thicket	Yes	No	Yes
WD5	Lowveld sour bushveld	Yes	Yes	Yes
WD6	Thicket	Yes	Yes	Yes
WD7	Savanna	Yes	No	No
WD8	Savanna	Yes	No	No
WD9	Mixed woodland	Yes	Yes	Yes
WD10	Malelane mountain bushveld	Yes	Yes	Yes
WD11	Malelane mountain bushveld	Yes	Yes	Yes
WD12	Mopane woodland	Yes	No	No
WD13	Phalaborwa sandveld	Yes	Yes	Yes
WD14	Savanna	Yes	Yes	Yes

**TABLE 5:** DNA content as measured by Qubit Fluorometric Quantification before sequencing, number of input sequences and number of sequences remaining in the analysis after each processing step for all samples.

Sample	DNA content (ng)	Sequences remaining after specified processing step				Final number of ASVs detected
		Input sequences	Filtering and trimming	Denoising	Non‐chimaeric	
WD1	221.33	69,107	5707	5200	5120	10
WD2	170.74	120,157	2646	2488	2488	8
WD3	255.66	23,609	1834	1239	1239	6
WD4	105.16	479,534	77,981	67,670	67,605	3
WD5	231.46	65,748	5318	5066	5066	6
WD6	280.16	28,583	1939	1897	1897	3
WD9	280.76	87,614	7415	6626	6626	8
WD10	109.14	73,887	9894	8901	8901	15
WD11	359.04	53,859	4469	4358	4358	6
WD13	111.76	148,006	10,633	9400	9400	9
WD14	311.552	120,787	11,930	10,422	10,422	15

After all filtering steps were completed, analysis using the DADA2 pipeline returned 308 ASVs between all samples. Of these ASVs, 178 were not assigned taxonomic classifications from our custom database, but the other 130 ASVs were assigned taxonomic classifications. Of these 130 ASVs, contaminant DNA was removed, and 59 ASVs with a percent identity match above 95% were used in the final assessment for prey species composition in each landscape type (Figure 4). The final number of ASVs in each sample is also given in Table 5 (the same ASV may be detected in multiple samples). The prey species composition was determined per landscape type by excluding host DNA and calculating each prey species' relative read abundance (RRA) out of the total prey species identified per landscape type. The specific identifications made per sample are listed in Table A2, separated by identifying marker.

## 3 Results

### 3.1 Stable Isotope Analysis

**FIGURE 2:**
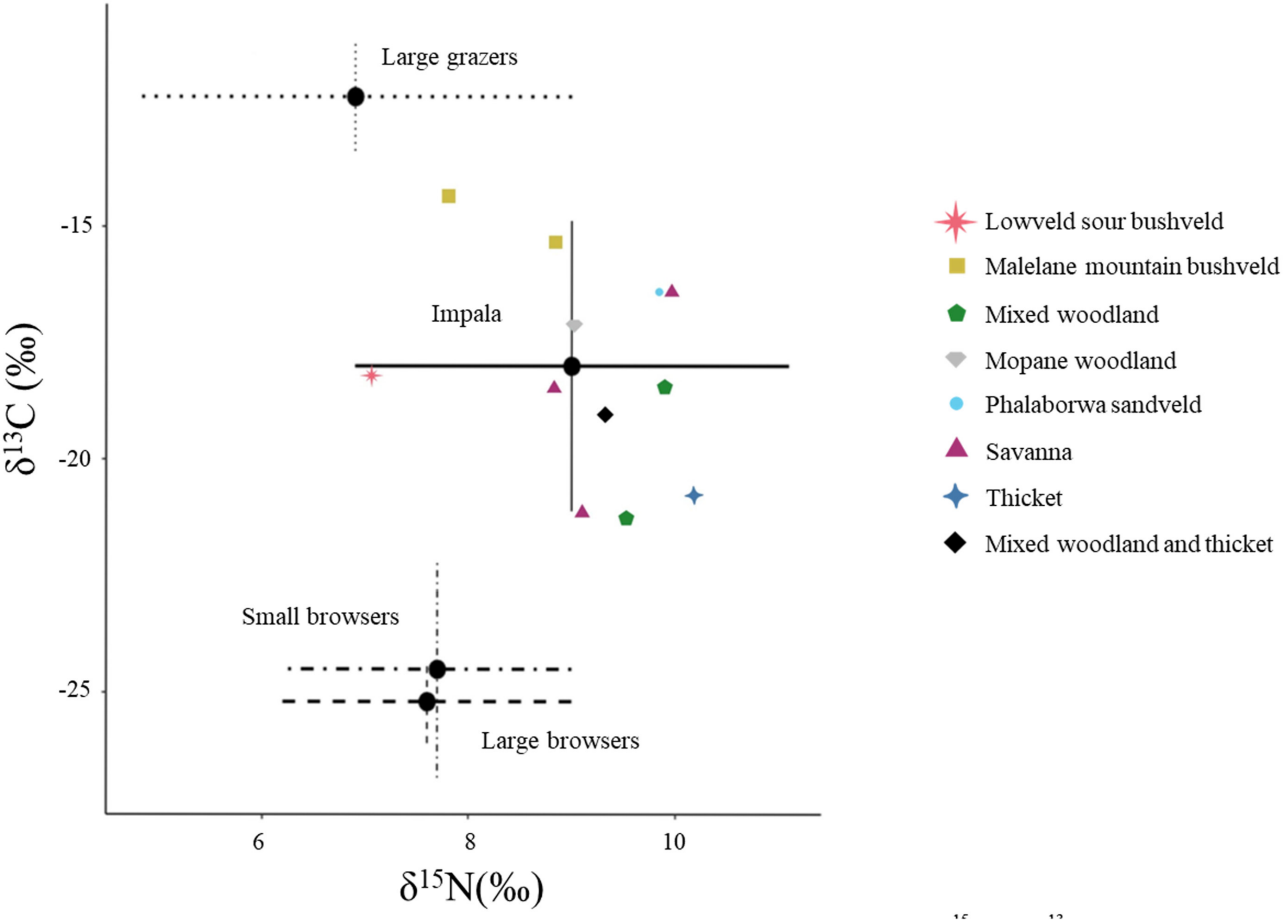
Isospace biplot showing the distribution of faecal samples according to their δ^15^N and δ^13^C isotope values, and where they cluster according to constituent prey types. Prey types are shown with large black circles (mean) and lines of varying types (solid, dotted, dashed and patterned for impala, large grazers, large browsers and small browsers respectively) for standard deviation. The last symbol of a black diamond has been used to represent two samples from different landscape types with identical isotope values.

**FIGURE 3:**
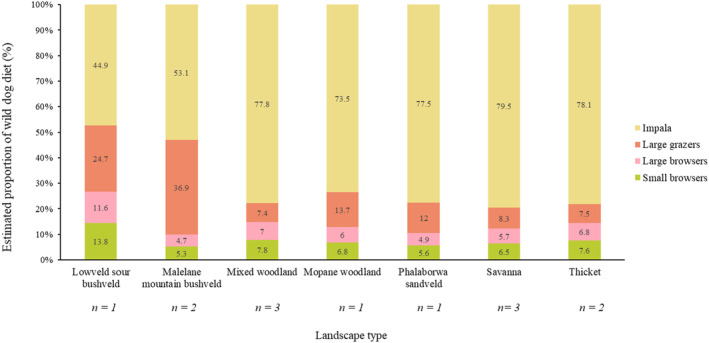
Stacked bar chart representing the mean percentage of each prey group present in wild dog diet across landscape types in the KNP. Data labels are provided with the percentage contribution of each prey type to total diet. Sample sizes of the faecal samples collected in each landscape type are represented (*n*) under each landscape type.

Carbon and nitrogen isotope ratios were measured for 13 of the 14 collected samples (Table 4). One sample could not be analysed due to insufficient faecal material. MixSIAR provided Gelman‐Rubin and Geweke diagnostics to test the validity of the model. The two diagnostic tests inform on whether MCMC chains have converged or not. Generally, the Gelman‐Rubin diagnostic value should be less than 1.05. For the analysis, all variables were less than 1.05. The Geweke diagnostic is a standard *z*‐score, so about 5% of variables are expected to be outside of ±1.96 (out of 20) in each chain. These requirements were met for all three chains in the analysis. The proportion of each prey type contribution to wild dog diet was provisionally modelled using an isospace biplot (Figure 2) which plots each datapoint according to their δ^15^N and δ^13^C values and shows around which prey groups the datapoints are clustered. The isospace biplot showed the majority of datapoints clustering around the impala prey source, with some spread towards large grazers. The data points exhibited almost no clustering around the small browser and large browser source prey points. The mean proportion of each prey type's contribution to wild dog diet across landscapes is visualised in a bar chart (Figure 3).

Impala formed the majority of prey consumed by the wild dogs across all landscape types, and over half the prey consumed in all landscapes apart from the Lowveld sour bushveld. The Lowveld sour bushveld and Malelane mountain bushveld reported the two lowest proportions for impala in wild dog diet (44.9% and 53.1% respectively), and for both landscape types the next biggest prey group were large grazers (24.7% and 36.9% respectively), representing a large difference compared to the proportion of large grazers in the five other landscape types. Small browsers were shown to make up a small percentage of wild dog diet across all landscape types, with only Lowveld sour bushveld having more than 10% of total diet made up by small browsers (13.8%). Large browsers were also best represented in Lowveld sour bushveld (11.6%) whereas all other landscape types had less than 10% of the total diet made up by large browsers.

### 3.2 Metabarcoding

**FIGURE 4:**
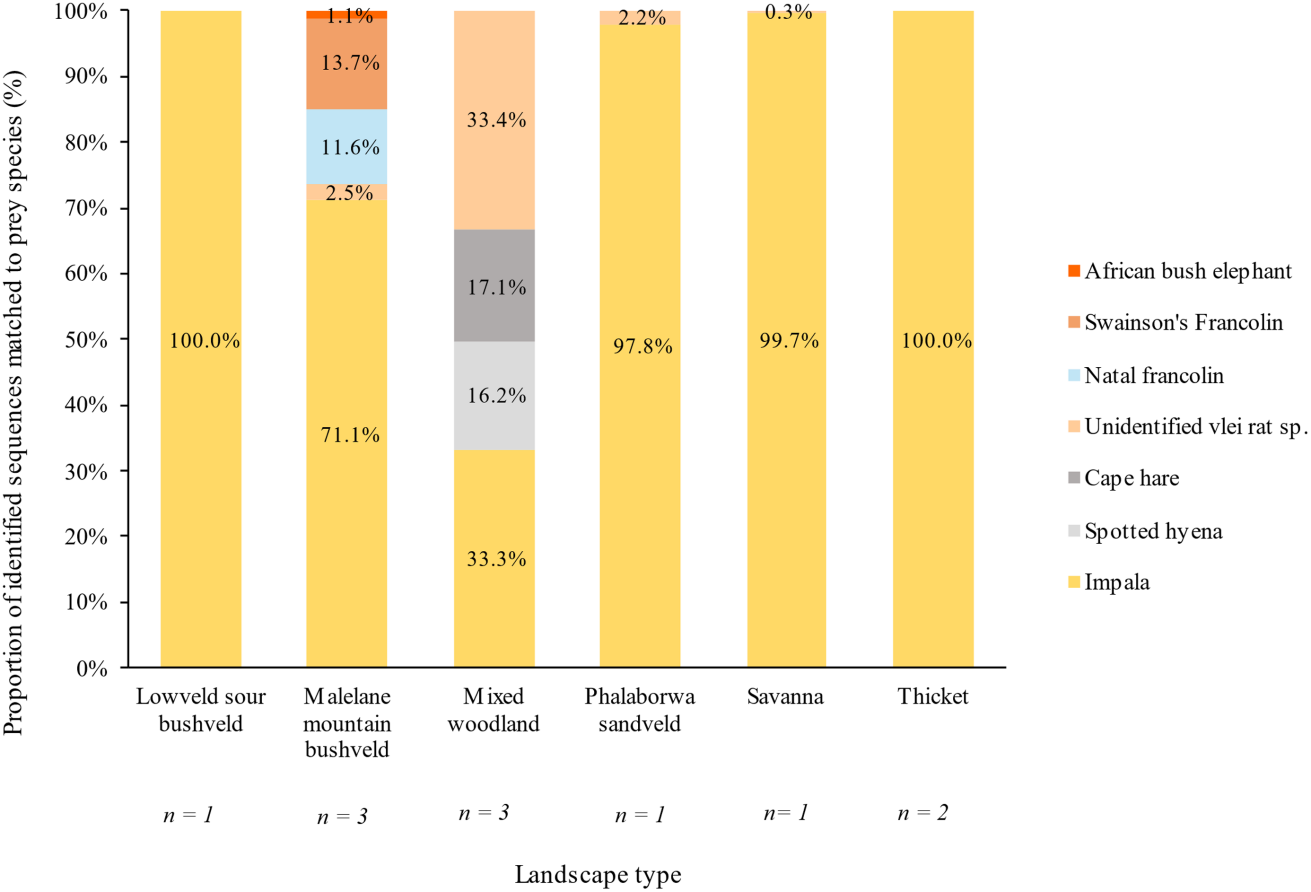
Stacked bar chart showing the proportion of identified sequences matched to prey species in each landscape type. Data labels were added which show the percentage of each prey species contribution to the diet composition in each landscape type.

**FIGURE A1:**
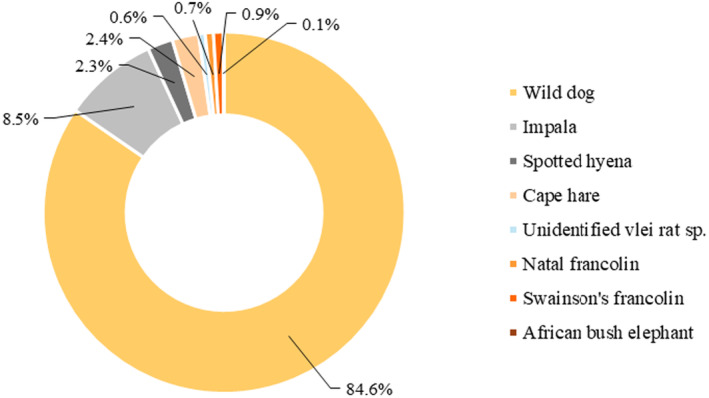
Donut chart representing the overall species proportions across all samples, with data labels indicating each prey species percentage makeup for all samples.

Overall, 8 taxa were identified, in total 7 prey taxa. Wild dog host DNA was overrepresented in almost all landscape types and formed the greatest proportion of all identified species (Figure A1). The proportion of wild dog sequences identified was over 50% in each landscape type. Wild dog was not included in the prey species composition graph (Figure 4) as it is not a prey species, and this allowed for better resolution of the smaller prey proportions.

Impala made up the major identified prey species in all landscapes, with only impala being identified in the Lowveld sour bushveld and thicket samples and forming almost all identified prey sequences in the Savanna save for a 0.3% proportion assigned to an unidentified vlei rat species. Malelane mountain bushveld was the most species rich landscape type, as well as the landscape type with the most samples collected therein. Two francolin species, Swainson's francolin (*Pternistis swainsonii*) and the Natal francolin (*Pternistis natalensis*) contributed 13.7% and 11.6% respectively to the species composition in the Malelane mountain bushveld. Trace vlei rat and African bush elephant (hereafter referred to as elephant) (*Loxodonta africana*) DNA made up the last two prey species identified for the Malelane mountain bushveld. The mixed woodland landscape type was the second most species rich and the only landscape type within which spotted hyena (hereafter hyena) (*Crocuta crocuta*) and the Cape hare (*Lepus capensis*) were identified. The unidentified vlei rat species and impala contributed a similarly large proportion of the identified species in mixed woodland, with 33.4% and 33.3% respectively. In the Phalaborwa sandveld, impala made up the largest proportion of diet in this landscape type at 97.8%, with the unidentified vlei rat species making up the rest of the diet composition.

For all samples pooled together, wild dog formed the greatest proportion of identified sequences at 84.6%, with impala following as the next largest proportion at 8.5%, then the Cape hare and hyena with 2.4%, 2.3% respectively. The unidentified vlei rat species, francolin species and elephant all contributed less than 1% of identified sequences across all samples (Figure A1).

## 4 Discussion

Most remaining wild dogs in Africa inhabit woodland and woodland savanna habitats (Hubel et al. 2016) and are generally believed to primarily hunt medium‐sized ungulates weighing between 15 and 200 kg (Creel and Creel 1995). Previously reported observational data suggested that wild dogs prey predominantly upon impala, with the greater kudu, steenbok, grey duiker, bushbuck and reedbuck making up small percentages of the remaining prey species (Creel, Mills, and McNutt 2004). These observations have resulted in wild dogs being considered specialist foragers with a narrow niche breadth (Vissia et al. 2023). Wild dogs are also thought to be rate‐maximising optimal foragers (Crossey et al. 2021; Mills 1992; Mills and Gorman 1997; Reich 1984), where they specialise in hunting the most abundant medium to large‐sized ungulates available in an area, and forgo opportunistic hunts (Crossey et al. 2021; Fuller et al. 1992). In most South African reserves, including the KNP, impala is the most abundant prey species in this category (Mills and Gorman 1997) and is widely spread and common across the Park (Chirima, Owen‐Smith, and Erasmus 2012). Both stable isotope analysis and metabarcoding analysis predicted across all landscape types that impala would constitute the majority of wild dog diet, which is consistent with these observations on prey abundance and wild dog hunting patterns (Davies‐Mostert, Mills, and Macdonald 2013). Hunting methods and diet preferences however are expected to change across landscape types for wild dogs, such as where long distance pursuits are observed in grass plains habitats in East Africa while less co‐operative shorter pursuits are characteristic of dense woodland and thicket habitats (Hubel et al. 2016). The sample sizes used in this study limit the degree to which the wild dog diet can be inferred, both with the stable isotope analysis and the metabarcoding analysis. For example, in the stable isotope analysis, dietary results from the Lowveld sour bushveld, Phalaborwa sandveld and savanna are derived from only one sample each, which reduces their power for making inferences on dietary preference as a single sample likely originates from only one dog. However, the results still demonstrate the promising application of metabarcoding coupled with stable isotope analysis for future studies that are allowed more intense and focussed sampling efforts. Suggestions for explaining the dietary patterns and landscape differences observed with these small sample sets are still given below and are encouraged to be further investigated.

Deviations to the general trend of impala forming the majority of wild dog diet do occur. Based on stable isotope data, this was observed in the Lowveld sour bushveld and Malelane mountain bushveld, where large grazers made up a significant part of wild dog diet after impala. Historically, impala have been recorded as less abundant in these far south‐eastern parts of the Park (Chirima, Owen‐Smith, and Erasmus 2012). This could be attributed to the sour grasses in these regions which are less palatable to impalas as compared to large ungulates (Redfern, Ryan, and Getz 2005).

The greater contribution of large grazers to wild dog diet in the Lowveld sour bushveld and Malelane mountain bushveld may also be attributed to predator competition dynamics. Lions frequent the habitats of their most favoured prey (e.g., buffalo (Mills and Shenk 1992)) and are found more commonly in the *Combretum* bushveld, *Acacia* thickets and *Marula* savanna, not the Lowveld sour bushveld and Malelane mountain bushveld (Mills and Gorman 1997). Wild dogs avoid areas that lions frequent due to the high associated mortality that lion predation has on wild dogs (Mills and Gorman 1997). Wild dogs also more easily make successful kills on larger prey in lion‐free areas where they aren't chased off (van Dyk and Slotow 2003). Impala have been shown to be underrepresented in lion dietary analyses (Funston and Mills 2006) so the large contribution that impala made to wild dog diet across all landscapes may be explained by lack of competition, as well as their abundance.

Despite the sour grass types, grazers contributed more to the wild dog diet in Lowveld sour bushveld and Malelane mountain bushveld than both large browsers and small browsers in the SIA results. In fact, the stable isotope results indicated that browsers are generally underrepresented across all landscape types. This stands in contrast to the conclusions that Crossey et al. (2021) drew using hair samples; that small browsers contribute a larger part to wild dog diet than previously thought.

Broadly comparing the SIA of the faecal samples in this study with the hair sample results of (Crossey et al. 2021), impala were overrepresented, small and large browsers were underrepresented and large grazers were somewhat similar between the two studies for Lowveld sour bushveld, mixed woodland and Malelane mountain bushveld, but underrepresented in thicket. These four landscape types are the only directly comparable types as Crossey et al. (2021) excluded the other landscape types from their results based on small sample sizes.

Differences in the stable isotope results delivered by the faecal samples in this study and the hair samples collected by Crossey et al. (2021) may be accounted for by the larger sample sizes in (Crossey et al. 2021) or the nature of the sample types. Faecal samples may more accurately represent diet differences between landscapes as they capture short‐term information (Franz et al. 2023). For African wild dogs, gut passage time ranges from a minimum of 5.5 h to a maximum of 79.4 h, and so diet composition data obtained from faecal samples provides insight into prey preferences only for up to four days (Davies‐Mostert et al. 2010). A faecal sample collected in a given landscape type is more likely to have resulted from a wild dog that hunted in the same landscape type. (Crossey et al. 2021) cite (Marneweck et al. 2019) in their study, as these authors simultaneously monitored pack sizes for the Kruger National Park wild dogs while (Crossey et al. 2021) were collecting samples. Pack home ranges were determined to overlap multiple landscape types at the same time (Marneweck et al. 2019), indicating that SIA across longer time scales using hair samples may not be as accurate as analysis using faecal samples when resolving differences in diet across landscape types. The long‐term data provided by hair samples are more likely to represent actual prey preferences however, while faecal samples represent what was eaten in the last ~3 days (Davies‐Mostert, Mills, and Macdonald 2013) indeterminate of whether it was eaten due to preference, desperation or opportunity.

The metabarcoding results represent the first attempt at non‐invasive high‐resolution diet composition analysis for African wild dogs. Out of the fourteen samples that were obtained, only three failed to amplify, which may be explained by the age of the samples (Reddy et al. 2012). The large proportion of impala across almost all the landscape types according to the metabarcoding analysis is consistent with the literature on wild dog diet, and with the preliminary SIA diet composition prediction. The two landscape types with the most samples (Malelane mountain bushveld and the mixed woodland each with three samples) had the greatest variety of species, which again is likely linked to the fact that one sample represents only one dog, while multiple samples would presumably come from multiple dogs and thus be more species rich. The ability to accurately infer proportional diet contributions from the RRA of prey species is debated (Snider et al. 2022). Direct comparisons made between the proportion of food fed to an animal and the proportion of that food represented through metabarcoding analysis have shown the two values can differ significantly (Deagle et al. 2010). This may be due to the type of ingested material; muscle contains more mitochondria than other tissues, harder tissues persist better after digestion and some prey species have more copies of a marker than others (Snider et al. 2022). A sample that is better quality, with a higher DNA content, may also allow for better taxonomic resolution. Inferring diet proportion from RRA has however also proven reliable in metabarcoding analyses, particularly given large sample sizes (Sullins et al. 2018; Snider et al. 2022). Deagle et al. (2019) explain that although it has been common practice for metabarcoding diet investigations to remain conservative and report their findings as presence/absence data, RRA data can reliably and accurately depict population‐level dietary patterns when biases in DNA recovery are acknowledged. Furthermore, the authors argue that to equate prey species read abundances that are very low (say 100) and very high (say 10,000) and make no further investigations into the degree to which each species might contribute to the host's diet limits the ecological benefits that DNA metabarcoding data provides (Deagle et al. 2019).

Wild dogs have been documented before to pursue birds such as crested francolin and small mammals such as scrub hares (Davies‐Mostert et al. 2010) and cane rats (Vogel, Somers, and Venter 2018) and similar results were delivered in our samples with the identification of the vlei rat species, Cape hare, Natal francolin and Swainson's francolin. The presence of small mammal and bird species in the wild dog's diet may indicate a degree of individualism in the dog's behaviour, where small meals are opportunistically eaten and unlikely to be shared with the rest of the pack, which is not often considered in this highly social and co‐operative carnivore.

Wild dog and hyena share the same habitats (Bucci, Nicholson, and Krausman 2022), and wild dogs have been shown not to avoid hyenas spatially or temporally as they do lions (Darnell et al. 2014). The rivalry between African wild dogs and hyenas is well‐known (Bucci, Nicholson, and Krausman 2022; Carbone, Du Toit, and Gordon 1997; Creel and Creel 1998; Estes and Goddard 1967; Woodroffe 2011) and although hyenas are known to kill wild dogs (Creel 2001) cases where wild dogs have killed or eaten hyenas have not been recorded in the literature. At the time of their study, Estes and Goddard (1967) noted that although mobbing behaviour and harassment were commonly aimed towards hyenas from packs of wild dogs, they had never observed the dogs injuring or killing a hyena. The proportion of hyena identified as a possible prey species for wild dogs from the metabarcoding analysis could therefore be the first evidence of wild dogs acting with equal ferocity towards hyenas. Kamler et al. (2007) reported that wild dogs in Venetia Limpopo Nature Reserve would opportunistically kill and eat black‐backed jackal, or at the very least harass them if they crossed paths. It is therefore possible for wild dogs to prey upon other carnivores, though this may be less likely for hyena which are larger than wild dogs as compared to jackals. Through their shared use of the same habitats and the close contact that they come into during conflicting events such as kleptoparasitism (Carbone, Du Toit, and Gordon 1997), it is however not unlikely that environmental contamination could occur with hyena hair, urine or other faecal matter being collected with the wild dog faeces. Additionally, coprophagy may lead to identification of a ‘prey species’ where the host has ingested faecal matter which identifies the defaecating animal (Waggershauser et al. 2022). Wild dogs have not been reported to eat hyena faeces though autocoprophagy does occur in the species, and thus it should not be ruled out as another possible source of the hyena identification. Environmental contamination is an acknowledged challenge in metabarcoding dietary analyses (Alberdi et al. 2019; Ando et al. 2020) and this could potentially account for the detection of hyena, which has not previously been recognised as a part of wild dog diet.

Wild dogs rarely scavenge (Creel and Creel 1995) though the detection of elephant DNA in the metabarcoding results could be attributed to such behaviour. While efficient predators, wild dogs would not take on full grown elephants. As elephant DNA was only detected in one sample, it likely represents a singular, opportunistic, scavenging event.

There are five vlei rat species in southern Africa and the custom database of possible prey species included sequences from four of them; the Angoni vlei rat (*Otomys angoniensis*), the southern African vlei rat (*Otomys irroratus*), the laminate vlei rat (*Otomys laminatus*) and Sloggett's vlei rat (*Otomys sloggetti*). No 12S and 16S mitochondrial sequences were available for Saunder's vlei rat (*Otomys saundersiae*) and only partial sequences, of the 12S gene only, were obtained for the Angoni vlei rat and the laminate vlei rat. Sloggett's vlei rat was the identified vlei rat species in these samples, though the classification was maintained at a genus level. The assignment of Slogget's vlei rat is unexpected as the distribution of Sloggett's vlei rat does not fall near the KNP and the species is endemic to the high‐altitude, cold areas of the southern Drakensberg and Maluti mountains (Schwaibold 2005). More likely vlei rat species with distributions that do fall nearer to the KNP are the Angoni vlei rat or the laminate vlei rat (Taylor et al. 2016). The failure of these vlei rats to match with the same sequence that Slogget's vlei rat did may be attributed to the limited existing data on their mitochondrial sequences. It is more likely that an improved database of 12S and 16S sequences for the Angoni and laminate vlei rat would produce more fitting results considering the ecology and distribution of these two species.

The diet composition prediction made using SIA and metabarcoding is generally in agreement, with impala acting as the main diet component. However, our metabarcoding results add value to the SIA in their identification of the presence of other prey species less commonly (or not at all) reported in the literature for African wild dogs. Modifications and improvements may be made to increase species identifications. Alternative gene regions such as the COI or cytochrome *b* may be chosen (Hebert et al. 2003), though COI has been shown to perform poorly in comparison to 12S primers (Collins et al. 2019). Less degenerate primers could also be used to increase primer specificity, particularly where the reference database of prey species is small and localised (Elbrecht, Hebert, and Steinke 2018). Most importantly, improved prey species DNA databases are needed, and host DNA amplification should be blocked (such improvements can increase identified prey taxa by more than 60% (Shehzad et al. 2012)). Increased sample sizes and rigorous sampling procedures would also benefit dietary inferences. The quality and quantity of DNA in a faecal sample have been shown to decrease significantly with prolonged exposure to direct sunlight (Reddy et al. 2012) and fresh samples (less than three days old) are critical to ensure good quality DNA and effective downstream analysis (Reddy et al. 2012). Non‐optimal sampling techniques can increase the difficulty of the already challenging task of extracting and amplifying fragmented DNA that has passed through the gut from faecal samples (King et al. 2008) and may be the reason why three samples in this study failed to amplify.

This study has proven the validity of DNA metabarcoding for the dietary analysis of African wild dogs. The preliminary results indicate DNA metabarcoding to be a viable and insightful addition to investigations into wild dog diet, especially given the adoption of the abovementioned improvements and modifications. Metabarcoding approaches may be avoided in diet composition analyses by some due to the costs of molecular analysis; however, some of those costs can be mitigated by following effective protocols from the initial process of sampling until the final process of bioinformatics. For example, inhibiting the amplification of host or contaminant DNA (De Barba et al. 2014) and multiplexing samples so that sequencing may be conducted in one run for large datasets (Coissac, Riaz, and Puillandre 2012) improves the efficiency and reduces the cost of the DNA amplification and sequencing processes.

By inspecting the diet of an endangered carnivore such as the African wild dog, particularly that of an unmanaged self‐sustaining population, conservation efforts for the species may be enhanced. The aforementioned benefits of improved nutritional protocols, conflict mitigation and ecosystem health indicators are relevant for African wild dogs. A large number of dogs in South Africa are part of the managed metapopulation or the free‐roaming population, both of which are managed to some degree by organisations such as the Endangered Wildlife Trust (EWT) or the Waterberg Wild Dog Initiative (WWDI). These organisations may hold dogs captive for some time to aid translocations or supplement their hunting in the course of conflict mitigation and ecotourism strategies. These endeavours may be helped by enhanced data on what wild dogs eat in the wild. For example, our results suggest a greater proportion of small mammals in wild dog diet than previously thought. These species may be easier to acquire and feed to captive dogs than large buck species. This would also aid decision‐making in where to translocate dogs to and from. Additionally, when sampling efforts are large, metabarcoding of faecal samples may give an indication of both host and prey species distributions, abundances and shifts which are particularly vital for the wide‐roaming and declining African wild dog (Shehzad et al. 2012).

As this is the first study to utilise a metabarcoding approach in assessing wild dog diet, the prospects for future studies, applications in other elusive carnivores and the identification of novel prey species are promising.

## Author Contributions

**Bridget C. O'Connor:** conceptualization (equal), data curation (equal), formal analysis (equal), investigation (equal), methodology (equal), visualization (equal), writing – original draft (equal). **Bruce Crossey:** conceptualization (equal), data curation (equal), formal analysis (equal), investigation (equal), methodology (equal), project administration (equal), resources (equal), supervision (equal), writing – review and editing (equal). **Grant Hall:** conceptualization (equal), data curation (equal), formal analysis (equal), investigation (equal), methodology (equal), resources (equal), writing – review and editing (equal). **Andre Ganswindt:** conceptualization (equal), funding acquisition (equal), project administration (equal), resources (equal), supervision (equal), writing – review and editing (equal). **Carel J. Oosthuizen:** conceptualization (equal), data curation (equal), formal analysis (equal), funding acquisition (equal), investigation (equal), methodology (equal), project administration (equal), resources (equal), supervision (equal), writing – review and editing (equal).

## Conflicts of Interest

The authors declare no conflicts of interest.

## Data Availability

Data is available on Dryad Digital Repository at http://datadryad.org/stash/share/b0xr3LfSnfsFiJNnZQ3AN0‐De4jtypvUbmBvKJ84T7g.

## Appendix 1

**TABLE A1:** List of possible prey species, host species or contaminating species included in the custom database of 12S and 16S gene sequences.

Species		16S	12S	Species		16S	12S
*Aepyceros melampus*	Impala	Y	Y	*Otomys angoniensis*	Angoni vlei rat	N	Y
*Aethomys chrysophilus*	Red veld rat	Y	Y	*Otomys irroratus*	Southern African vlei rat	Y	Y
*Alcelaphus lichtensteinii*	Lichtenstein's hartebeest	Y	Y	*Otomys laminatus*	Laminate vlei rat	N	Y
*Cephalophus natalensis*	Red duiker	Y	Y	*Ourebia ourebi*	Oribi	Y	Y
*Chlorocebus pygerythrus*	Vervet monkey	Y	Y	*Papio ursinus*	Chacma baboon	Y	Y
*Connochaetes gnou*	Black wildebeest	Y	Y	*Pelea capreolus*	Grey rhebok	Y	Y
*Connochaetes taurinius*	Blue wildebeest	Y	Y	*Phacochoerus africanus*	Warthog	Y	Y
*Damaliscus lunatus lunatus*	Tsessebe	Y	Y	*Potamochoerus larvatus*	Bushpig	Y	Y
*Equus quagga burchelli*	Burchell's zebra	Y	Y	*Raphicerus campestris*	Steenbok	Y	Y
*Equus zebra*	Mountain zebra	Y	Y	*Raphicerus sharpei*	Sharpe's grysbok	Y	Y
*Galago moholi*	Lesser bush baby	Y	Y	*Redunca arundinum*	Common reedbuck	Y	Y
*Giraffa camelopardis*	Giraffe	Y	Y	*Redunca fulvorufula*	Mountain reedbuck	Y	Y
*Hippotragus equinus*	Roan	Y	Y	*Sylvicapra grimmia*	Common duiker	Y	Y
*Hippotragus niger*	Sable antelope	Y	Y	*Syncerus caffer*	Buffalo	Y	Y
*Homo sapiens*	Human	Y	Y	*Thryonomys swinderianus*	Greater cane rat	Y	Y
*Kobus ellipsiprymnus*	Waterbuck	Y	Y	*Tragelaphus angasii*	Nyala	Y	Y
*Lycaon pictus*	Wild dog	Y	Y	*Tragelaphus oryx*	Eland	Y	Y
*Otomys sloggetti*	Sloggett's vlei rat	Y	Y	*Tragelaphus scriptus*	Bushbuck	Y	Y
*Oreotragus oreotragus*	Klipspringer	Y	Y	*Tragelaphus strepsiceros*	Kudu	Y	Y
*Otolemur crassicaudatus*	Thick‐tailed bush baby	Y	Y	*Crocuta crocuta*	Hyena	Y	Y
*Loxodonta africana*	African bush elephant	Y	Y	*Lepus capensis*	Cape hare	Y	Y
*Pternistis natalensis*	Natal francolin	N	Y	*Pternistis swainsonii*	Swainson's francolin	Y	Y
*Dendroperdix sepahena*	Crested francolin	Y	Y	*Cordylus vittifer*	Transvaal girdled lizard	Y	Y
*Felis nigripes*	Black footed cat	Y	Y				

**TABLE A2:** Table showing the species identifications made per sample and which marker the identified sequence is represented by.

Sample	Identification	Marker
WD1	Wild dog	12S
	Wild dog	16S
	Impala	16S
	Unidentified vlei rat species	12S
	Impala	12S
WD2	Wild dog	12S
	Wild dog	16S
	Spotted hyena	16S
	Lepus capensis	16S
WD3	Wild dog	12S
	Wild dog	16S
	Unidentified vlei rat species	12S
	Impala	12S
WD4	Wild dog	16S
	Impala	16S
WD5	Wild dog	12S
	Wild dog	16S
	Impala	12S
WD6	Wild dog	16S
	Wild dog	12S
WD9	Wild dog	12S
	Wild dog	16S
	Unidentified vlei rat species	12S
	Impala	12S
WD10	Wild dog	12S
	Wild dog	16S
	Natal francolin	12S
	Impala	12S
	African bush elephant	16S
	Unidentified vlei rat species	12S
	Swainson's francolin	16S
WD1	Wild dog	12S
	Wild dog	16S
	Impala	12S
WD13	Wild dog	12S
	Wild dog	16S
	Unidentified vlei rat species	12S
	Impala	12S
WD14	Wild dog	12S
	Wild dog	16S
	Unidentified vlei rat species	12S
	Impala	12S
